# Comparison of the postoperative analgesic effect for infiltration between the popliteal artery and the capsule of the posterior knee and that of periarticular multimodal drug injection in total knee arthroplasty: retrospective study in the immediate postoperative period

**DOI:** 10.1186/s43019-019-0025-z

**Published:** 2020-01-01

**Authors:** Dae-Won Jung, Won-Yong Shon, Seung-Suk Seo, Ok-Gul Kim, In-Seung Lee

**Affiliations:** 1 Department of Orthopedic Surgery, Busan Bumin Hospital, 59, Mandeok-daero, Buk-gu, Busan, South Korea; 2 Department of Orthopedic Surgery, Haeundae Bumin Hospital, 584, Haeun-daero, Haeundae-gu, Busan, South Korea

**Keywords:** Total knee arthroplasty, Periarticular multimodal drug injection, Adductor canal block, IPACK

## Abstract

**Background:**

The aim of this study is to compare the postoperative analgesic effect of infiltration between the popliteal artery and the capsule of the knee (IPACK) and the effect of periarticular multimodal drug injection (PMDI) in addition to adductor canal block (ACB) after total knee arthroplasty.

**Methods:**

Among patients who received total knee arthroplasty from June 2017 to December 2017, 50 who underwent ACB with additional IPACK and 50 who received ACB with additional PMDI were selected for this study. We compared the postoperative pain numerical rating scale (NRS), the number of times patient-controlled analgesia was administered and the amount administered, the total amount of opioids given, and complications associated with the procedure between the two groups.

**Results:**

NRS measured at rest and 45° knee flexion at days 1 and 2 after surgery was significantly lower in the IPACK group than in the PMDI group. The resting NRS measured at day 3 after surgery was also significantly lower in the IPACK group than in the PMDI group, and the NRS at 45° knee flexion measured from day 3 to day 5 showed a significant reduction in the IPACK group. No complications relating to the procedure occurred.

**Conclusions:**

IPACK may be a better option than PMDI for controlling acute phase pain in patients undergoing total knee arthroplasty.

## Introduction

Total knee arthroplasty is satisfactory for improving pain and recovery from arthritis, but many patients complain of postoperative pain [1, 2]. Postoperative pain is an important factor affecting the outcome of surgery that can make rehabilitation difficult and limit the range of motion of the joint [3]. Nonsteroidal analgesics, narcotic analgesics, patient-controlled analgesia, and periarticular multimodal drug injection (PMDI) have been used to relieve postoperative pain [4–6]. Recently, peripheral nerve blocks such as femoral nerve block (FNB), adductor canal block (ACB), and sciatic nerve block have been used to control pain after total knee arthroplasty [7–9]. FNB may provide effective pain control, but it may also cause weakness in the quadriceps muscle after surgery, causing a limitation in gait [7]. Meanwhile, ACB is effective in relieving pain without causing weakness in the quadriceps muscle [10]. However, ACB is less effective in relieving posterior knee pain [11]. To compensate for this, ACB is combined with sciatic nerve block and PMDI [12]. Recently, a procedure using ultrasound-guided local anesthetic infiltration between the popliteal artery and the capsule of the knee (IPACK) has been shown to provide significant posterior knee analgesia without affecting the common peroneal nerve [12]. IPACK can also be performed to reduce the pain in the posterior knee, with virtually no risk of injury to the nerves or blood vessels. To date, however, there is no comparative study between IPACK and PMDI on postoperative pain control after total knee arthroplasty in Korean patients. In this retrospective study, under the hypothesis that the analgesic effect of IPACK would be superior to that of PMDI, we compared the effectiveness of ACB and PMDI with that of ACB and IPACK in reducing pain after total knee arthroplasty.

## Materials and methods

### Study design

**Fig. 1:**
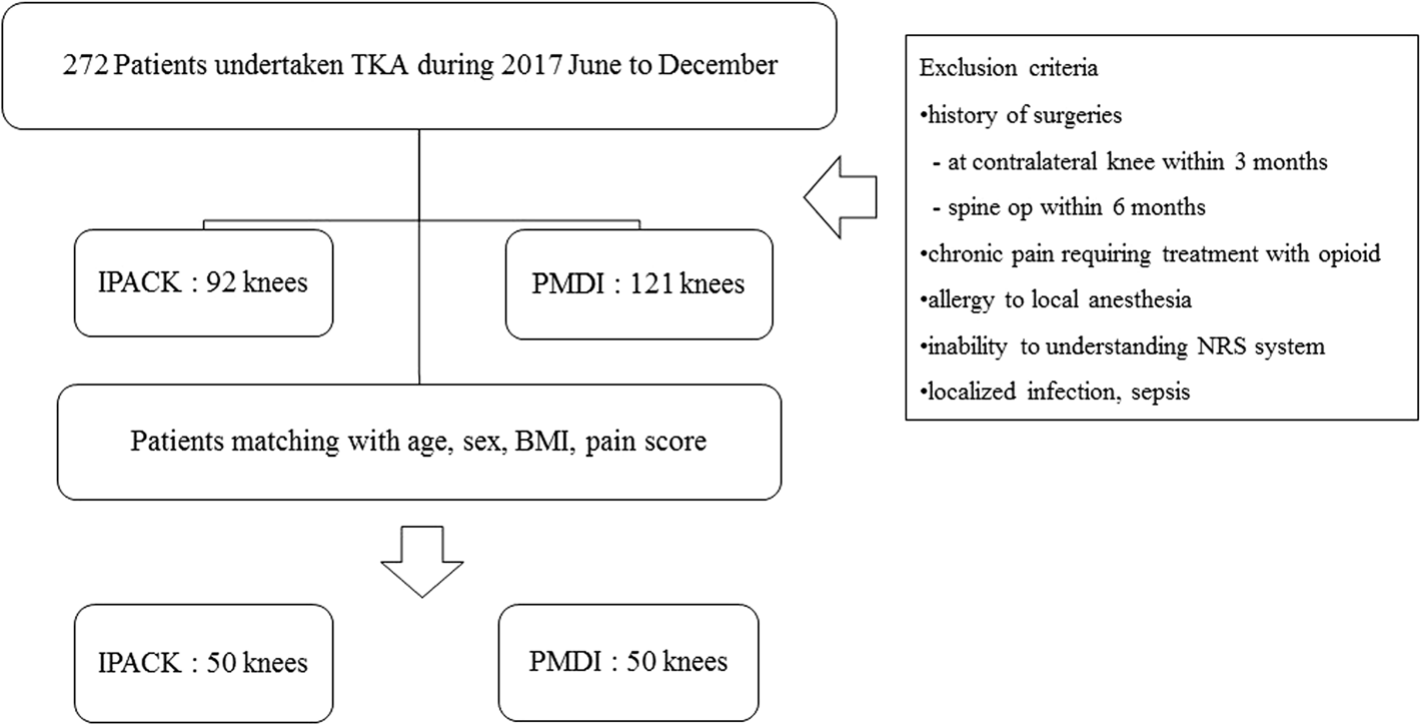
Patient screening and enrollment flowchart. BMI body mass index, IPACK infiltration between the popliteal artery and the capsule of the knee, NRS numerical rating scale, PMDI periarticular multimodal drug injection, TKA total knee arthroplasty

This study was approved by the Institutional Review Board (IRB) of Bumin Hospital (IRB 201905-BM-003)., Among patients who had undergone total knee arthroplasty by a single surgeon for degenerative arthritis of the knee from June to December 2017, 92 patients underwent IPACK (the IPACK group) and 121 patients underwent PMDI (the PMDI group) (Fig. 1). PMDI was used exclusively by us until August 2017 for controlling posterior knee pain after total knee arthroplasty. However, as our interest in IPACK grew, almost all subsequent patients received IPACK to control posterior knee pain after total knee arthroplasty. Patients who had undergone contralateral knee surgery within 3 months or spinal surgery within 6 months, patients with a history of allergic reactions to local anesthetics, patients with chronic pain requiring narcotic analgesics, patients who had difficulty identifying their pain numerical rating scale (NRS), patients who were not qualified for peripheral nerve block due to localized infection or sepsis and patients taking anticoagulation medication were excluded. Using the propensity score, 50 patients from each group whose body mass index (BMI; kg/m^2^), pain score and knee score did not show significant differences were selected, and their medical records were investigated retrospectively. All surgeries were performed under spinal anesthesia by an experienced anesthesiologist. All patients underwent ACB after spinal anesthesia. For ACB, an ultrasound survey was performed at the medial part of the thigh, halfway between the anterior superior iliac spine and the patella. We observed the femoral artery underneath the sartorius muscle, with the vein just inferior and the saphenous nerve just lateral to the artery. We ensured the catheter was correctly inserted in the saphenous nerve around the adductor canal using 2–3 mL saline solution. The catheter was then advanced 1 to 2 cm beyond the tip of the needle and 26 cm^3^ out of a total of 41 cm^3^ of solution made by mixing 40 cm^3^ 0.75% ropivacaine 150 mg and 1 cm^3^ dexamethasone 5 mg was inserted around the artery and was seen to have spread accurately.

### Technique for IPACK and PMDI

**Fig. 2:**
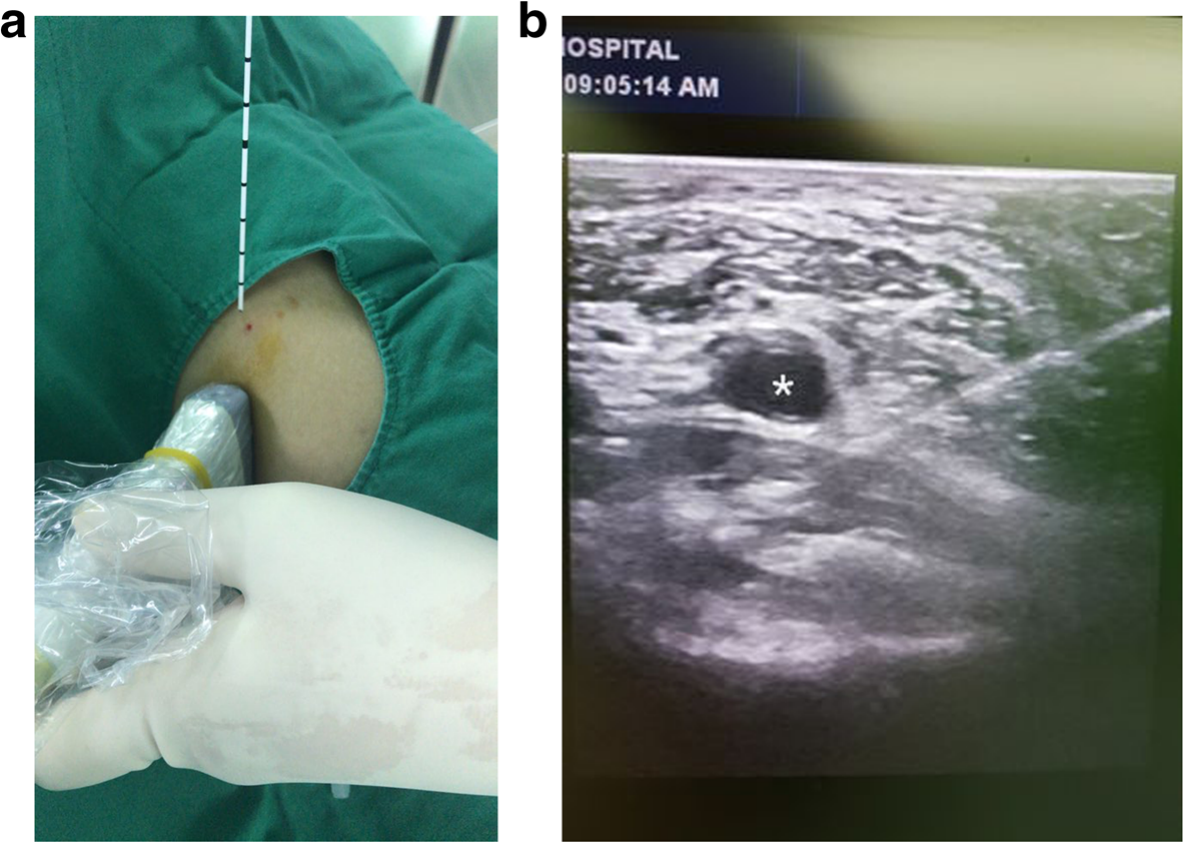
Infiltration between the popliteal artery and the capsule of the knee (IPACK) procedure at the knee joint using ultrasound guidance. **a** We approached via the medial side. **b** Under ultrasound guidance we observed the popliteal vessel and inserted medication in the space between this vessel and posterior capsule of the knee. *Popliteal artery

In the IPACK group, IPACK was performed using the remaining 15 cm^3^ of the 41 cm^3^ solution mentioned above (40 cm^3^ 0.75% ropivacaine 150 mg and 1 cm^3^ dexamethasone 5 mg) immediately after ACB. To perform the IPACK procedure, the patient’s knee was flexed to 90° and we approached via the medial side. Under ultrasound guidance, we observed the popliteal vessel and inserted medication into the space between this vessel and the posterior capsule of the knee [11] (Fig. 2).

**Fig. 3:**
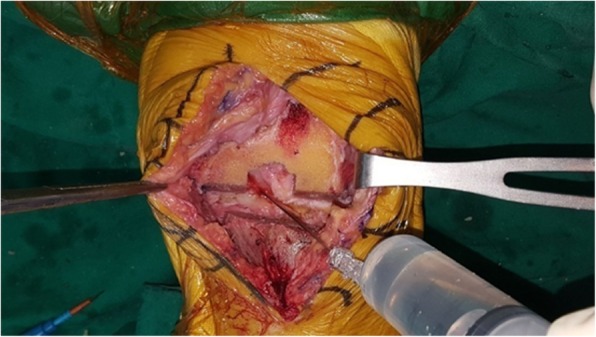
Periarticular multimodal drug injection (PMDI) at the posterior capsule before implant placement

In the PMDI group, 50 cm^3^ of a multidrug solution (20 cm^3^ 0.75% ropivacaine, 0.5 cm^3^ keromin 30 mg, 0.2 cm^3^ epinephrine 1 mg, 20 cm^3^ normal saline and 10 cm^3^ cefazoline 1 g) was locally and evenly injected to the suprapatellar pouch and quadriceps tendon, medial retinaculum, patellar tendon and fat pad, medial collateral ligament and medial meniscus capsular attachment, posterior cruciate ligament (PCL) tibial attachment, anterior cruciate ligament femoral attachment, lateral collateral ligament, lateral meniscus capsular attachment and lateral retinaculum before cementation for implant fixation [13, 14] (Fig. 3).

All patients in both groups received tri-compartment cemented knee arthroplasty with PCL-substituting implants under tourniquet control.

### Postoperative pain control

The patient-controlled analgesia was administered intravenously at 2 ml/h using a solution of 1 mg fentanyl citrate, 180 mg ketololac tromethamine, 8 mg zofran, and 100 ml normal saline until postoperative day 3. The patients were instructed to press a button on the patient-controlled analgesia system when the pain felt severe. Standard oral analgesics were administered every 12 h with celecoxib 100 mg, acetaminophen 325 mg, and tramadol hydrochloride 37.5 mg. If the patient complained of severe pain with an NRS of 7 or more, opioids (25 mg pethidine 0.5 ml intravenously) were administered.

### Outcome measures

The NRS of each group was measured by trained clinical nurses to determine the degree of postoperative pain. NRS was measured four times (06:00, 11:00, 16:00, and 21:00) until postoperative day 2, twice (06:00 and 16:00) on postoperative day 3, and once (06:00) on postoperative days 4 to 7 both at rest and with 45° flexion of the knee. In addition, the total number and amount of patient-controlled analgesics applied, along with opioid consumption and the duration of each procedure, were measured and the sequelae and complications of each method were compared.

### Statistical analysis

MedCalc (MedCalc 15.2.2 version, MedCalc Inc., Mariakerke, Belgium) was used for statistical analysis. The Kolmogorov-Smirnov test was used to determine whether the measured and calculated parameters were distributed correctly. An independent-samples *t* test was used to determine the significance of differences in continuous variables between groups. The chi-square test was used for correlations between two categorical variables. A *P* value less than 0.05 was taken to be statistically significant.

## Results

**Fig. 4:**
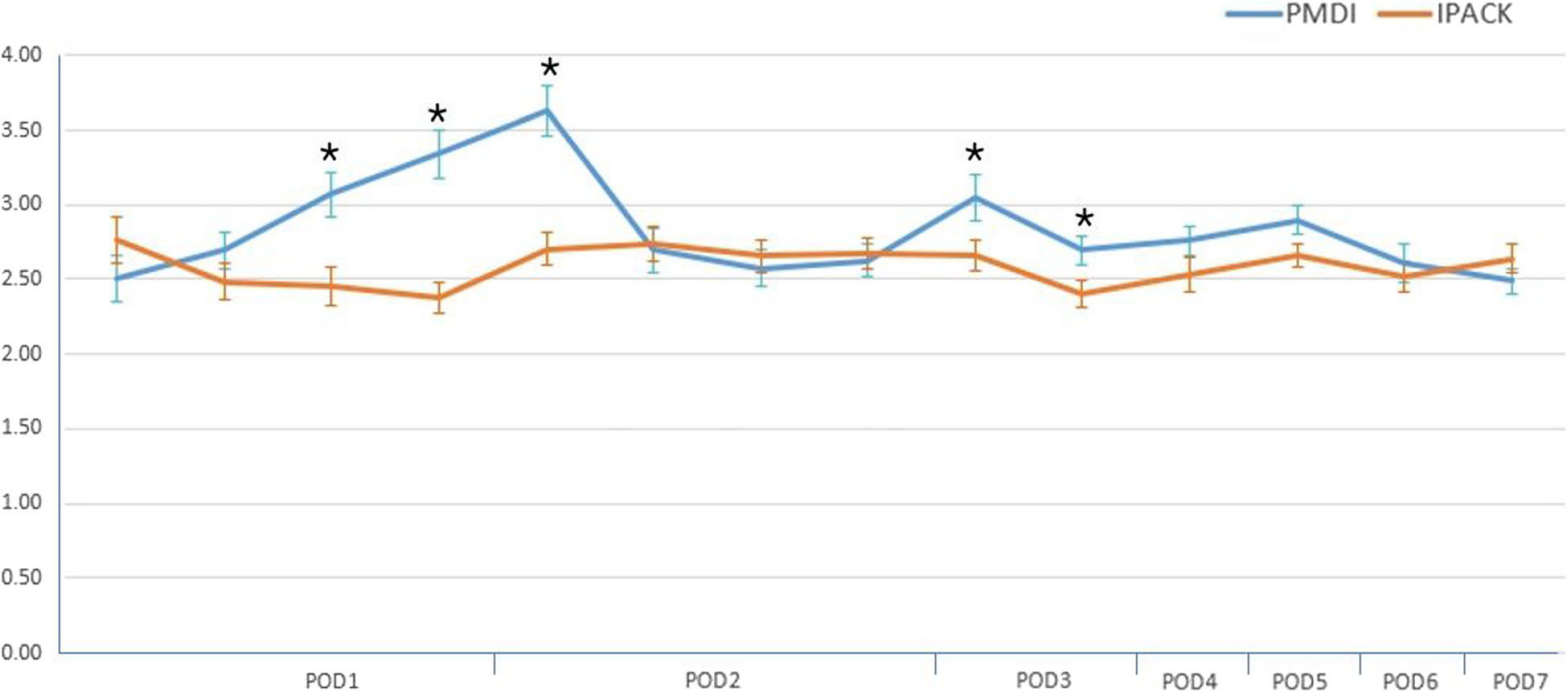
Comparison of the postoperative resting numerical rating scale between the two groups. *P < 0.05, versus IPACK. IPACK infiltration between the popliteal artery and the capsule of the knee, PMDI periarticular multimodal drug injection, POD postoperative day

**Fig. 5:**
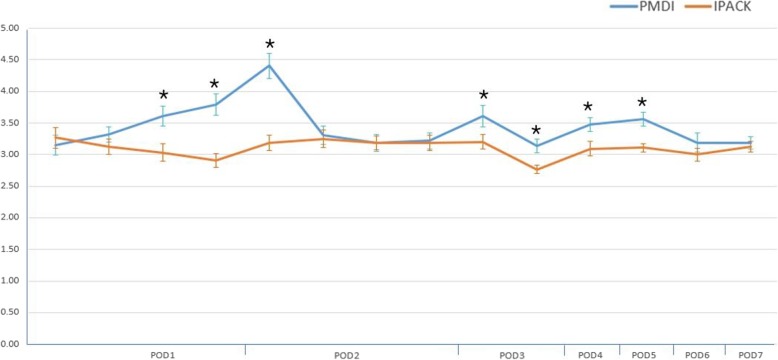
Comparison of postoperative 45° knee flexion numerical rating scale between the two groups. *P < 0.05, versus IPACK. IPACK infiltration between the popliteal artery and the capsule of the knee, PMDI periarticular multimodal drug injection, POD postoperative day

**Fig. 6:**
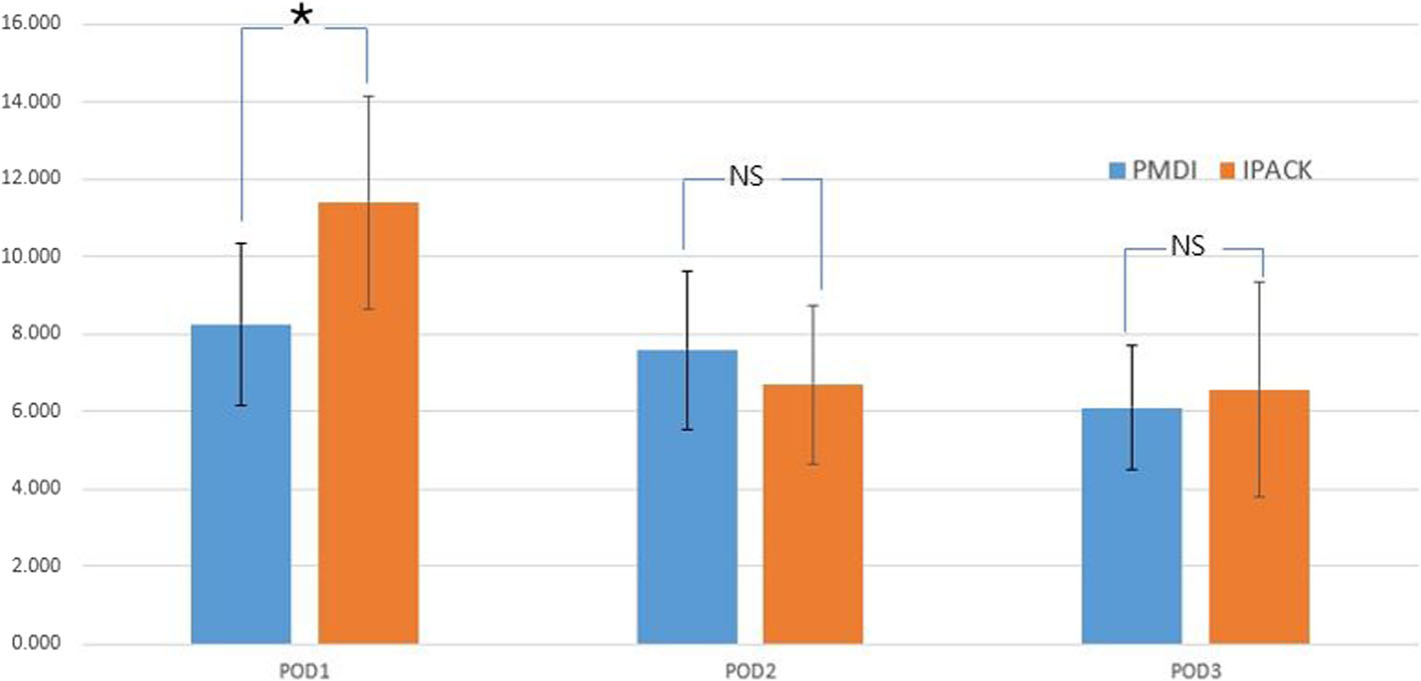
Comparison of the mean number of times that patients pushed their patient-controlled analgesia button between two groups. *P < 0.05. IPACK infiltration between the popliteal artery and the capsule of the knee, NS not significant, PMDI periarticular multimodal drug injection, POD postoperative day

**Fig. 7:**
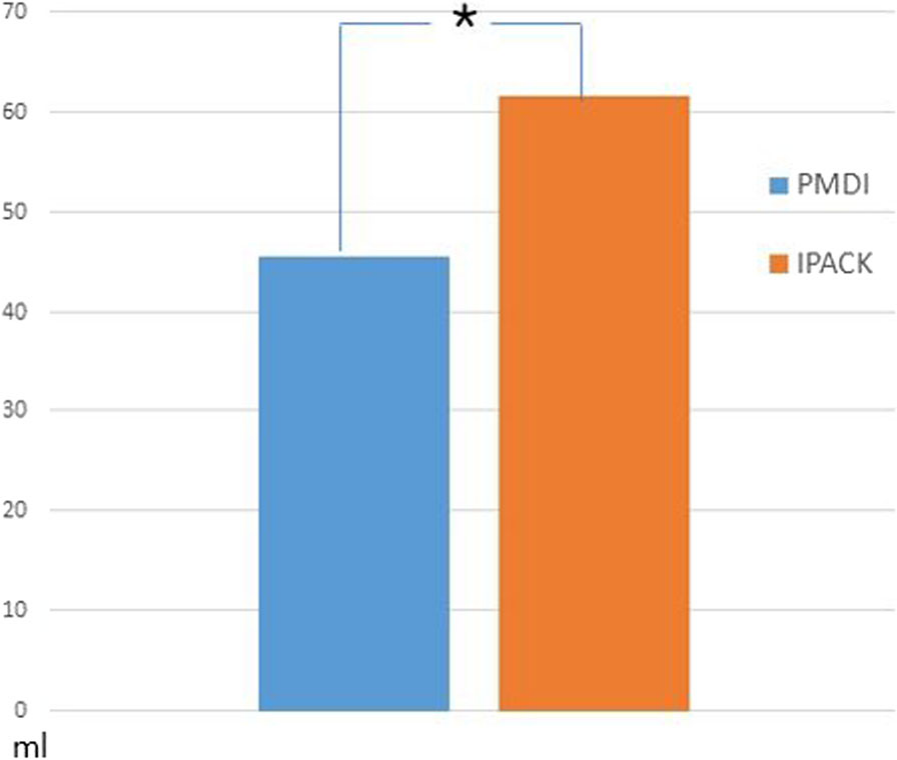
Comparison of total volume of patient-controlled analgesia between two groups. *P < 0.05. IPACK infiltration between the popliteal artery and the capsule of the knee, PMDI periarticular multimodal drug injection

**Fig. 8:**
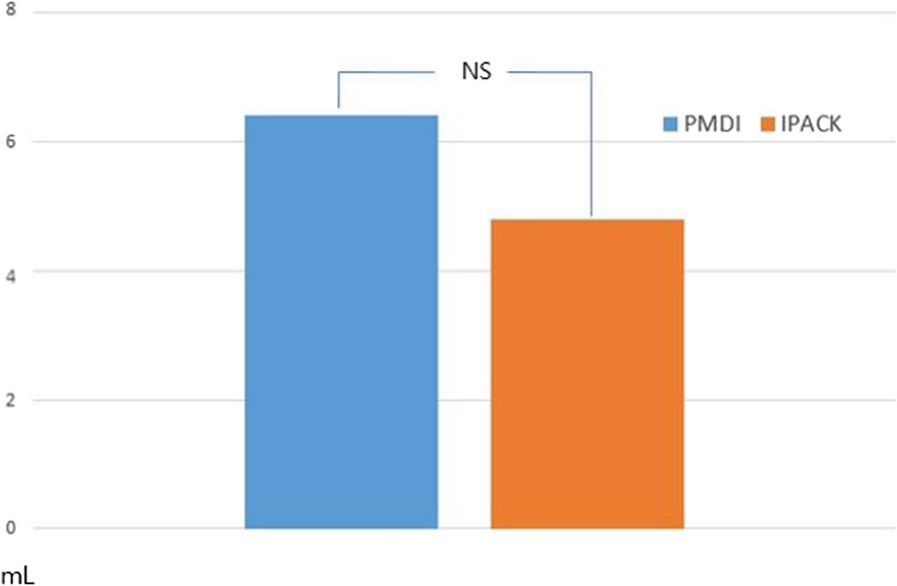
Comparison of total volume of opioid consumption between two groups. IPACK infiltration between the popliteal artery and the capsule of the knee, NS not significant, PMDI periarticular multimodal drug injection

**Fig. 9:**
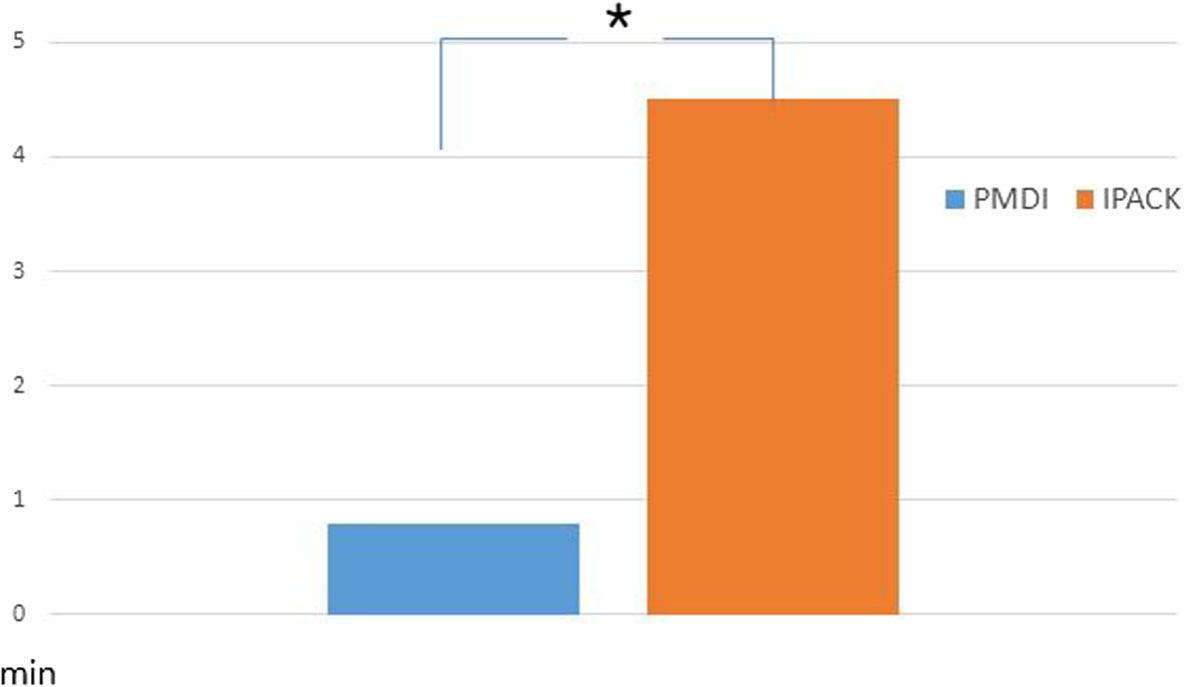
Comparison of the duration of procedure between two groups. *P < 0.05. IPACK infiltration between the popliteal artery and the capsule of the knee, PMDI periarticular multimodal drug injection

Of the 50 patients who underwent IPACK, two were male and 48 were female. Of the 50 patients who were treated with PMDI, all 50 were female. The mean age of the IPACK group was 69.3 years and the mean age of the PMDI group was 71.4 years. The mean BMI was 25.4 and 25.3 kg/m^2^ for the IPACK and PMDI groups, respectively, and the mean preoperative NRS was 7.4 and 7.3, respectively. The mean preoperative Knee Society Score (KSS) score was 52.6 for the IPACK group and 54.9 for the PMDI group (Table 1). These demographic data were not significantly different between both groups. At rest, the third and fourth NRS measurements on postoperative day 1, the first NRS measurement on postoperative day 2, and NRS on postoperative day 3 were significantly lower in the IPACK group than in the PMDI group (*P* = 0.022, *P* = 0.014, *P* = 0.01, *P* = 0.041 and *P* = 0.043, respectively) (Fig. 4). At 45° knee flexion, the third and fourth NRS measurements on postoperative day 1, the first NRS measurement on postoperative day 2, and NRS on postoperative days 3 to 5 were significantly lower in the IPACK group than the PMDI group (*P* = 0.031, *P* = 0.024, *P* = 0.008, *P* = 0.035, *P* = 0.039, *P* = 0.042 and *P* = 0.037, respectively) (Fig. 5). The number of times that patients pushed their patient-controlled analgesia button was significantly higher in the IPACK group on postoperative day 1, but showed no significant difference on postoperative days 2 or 3 (*P* = 0.027, *P* = 0.84 and *P* = 0.91, respectively) (Fig. 6). The total volume of additional patient-controlled analgesia was significantly higher in the IPACK group than the PMDI group (*P* = 0.013) (Fig. 7). The total volume of additional opioids was higher in the PMDI group than the PMDI group, but there was no statistically significant difference between the two groups (*P* = 0.059) (Fig. 8). The duration of the procedure was significantly longer in the IPACK group than in the PMDI group (*P* = 0.003) (Fig. 9). There were no procedure-related postoperative complications, such as generalized pruritus, dizziness, hypotension, hematoma or infection, in either group.

**Table 1:** Demographic and preoperative patient data

Characteristic	IPACK group	PMDI group	*P* value
Number of patients	50	50	
Gender (male/female)	2/48	0/50	0.516^a^
Age (years)	69.3 ± 7.5	71.4 ± 6.5	0.118^b^
Height (cm)	152.5 ± 6.5	153.1 ± 5.8	0.588^b^
Body mass index (kg/m^2^)	25.4 ± 3.1	25.3 ± 2.8	0.954^b^
Preoperative NRS	7.4 ± 2.3	7.3 ± 3.5	0.138^b^
Preoperative KSS^b^	52.6 ± 5.0	54.9 ± 6.5	0.093^b^
Preoperative knee flexion (°)	126.2 ± 5.7	127.1 ± 6.1	0.729^b^
Hypertension (%)	28/50 (56)	24/50 (48)	0.141^a^
Diabetes (%)	15/50 (30)	18/50 (36)	0.474^a^

Values are presented as mean ± standard deviation

*IPACK* infiltration between the popliteal artery and the capsule of the knee, *KSS* Knee Society Score, *NRS* numerical rating scale, *PMDI* periarticular multimodal drug injection

^a^Chi-square test; ^b^independent-samples *t* test

## Discussion

In the present study, NRS was significantly lower in the IPACK group at rest and with 45° knee flexion at days 1 and 2 after surgery than in the PMDI group. NRS measured 3 days after surgery also showed that the score was significantly lower in patients who underwent IPACK than in patients who underwent PMDI. The NRS at 45° knee flexion on postoperative days 3 and 5 was significantly lower in the IPACK group. Therefore, the IPACK group had their pain more effectively managed than the PMDI group. However, a controversy remains; the consumption of opioids was lower in the IPACK group, but the total volume of patient-controlled analgesia and the number of patient-controlled analgesia doses on postoperative 1 day was higher in the IPACK group. It is impossible to judge whether the pain improves because of IPACK or because of patient-controlled analgesia. Since patients can press the patient-controlled analgesia button regardless of the pain caused by a tourniquet or postoperative pain arising from any part of the knee, it is not appropriate to question the effectiveness of IPACK just because the patient-controlled analgesia button is pressed more times. We believe that more prospective studies with a bigger sample size are necessary.

Pain control after total knee arthroplasty has a remarkable effect on postoperative rehabilitation and clinical outcome as well as on patient satisfaction [15, 16]. Recently, peripheral nerve block has been widely used for postoperative pain management since there is no significant difference on pain control compared with epidural anesthesia, which is also effective for postoperative pain control, and it does not have the side effects of epidural anesthesia such as spinal hematoma formation, hypotension, dizziness, and systemic pruritus [17]. FNB, a type of peripheral nerve block, is effective in controlling pain after total knee arthroplasty, but it has the disadvantage of weakening the quadriceps muscle [7, 18, 19]. Meanwhile, ACB has recently become a popular pain control method because it does not show a significant difference in controlling pain compared with FNB and it does not cause weakness of the quadriceps muscle, making rapid rehabilitation possible [10, 20]. However, neither FNB nor ACB are very effective in relieving posterior knee pain [12, 21, 22]. Posterior knee pain is caused by the joint branch originating from the tibial component of the sciatic nerve originating from the obturator nerve [23]. To alleviate posterior knee pain, PMDI, sciatic nerve block and IPACK can be applied in combination [11]. PMDI can be performed easily and quickly, with theoretically no risk of injury to the nerves or blood vessels. However, direct injection into the knee joint has the disadvantage of possible infection [24]. In addition, PMDI can only be performed during surgery, and additional injections are impossible. Sciatic nerve block is another effective method to reduce posterior knee pain, but it can cause foot drop in 65–68% of cases [12, 25]. It may be difficult to differentiate patients with foot drop due to peroneal nerve injury; thus, the proper treatment time may be missed, and the risk of falls may increase. An alternative to sciatic nerve block is selective tibial nerve block in the popliteal fossa, which may provide posterior pain relief without causing foot drop, but can cause numbness and weakness in plantar flexion [12]; thus, it does not consistently avoid blockade of the common peroneal nerve. It is therefore necessary to develop anesthesia techniques that can control posterior knee pain without causing muscle weakness and numbness.

**Fig. 10:**
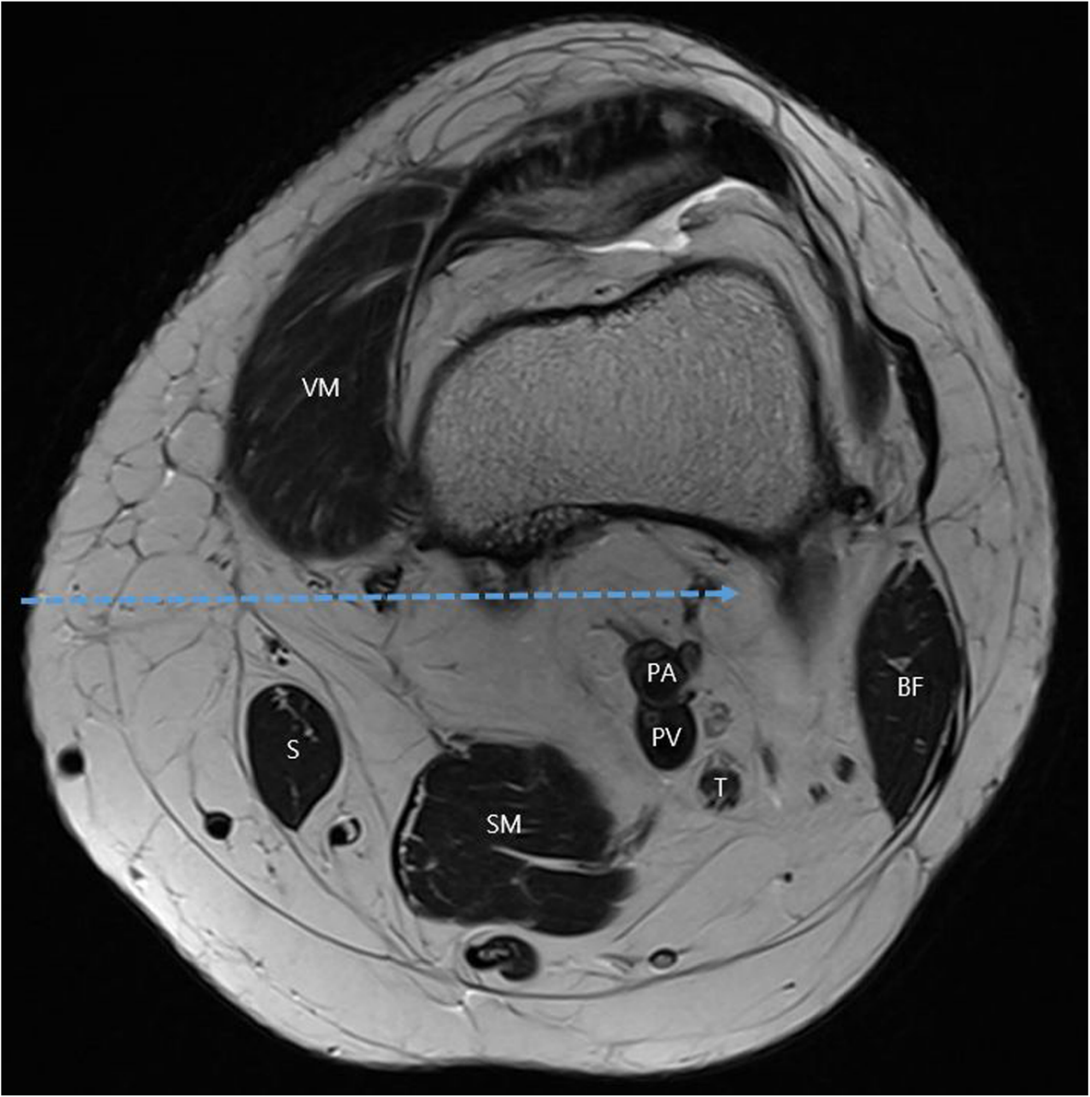
Cross-sectional anatomy of the thigh proximal to the left femoral condyles. The target tissue plane for infiltration between the popliteal artery and the capsule of the knee (IPACK) injection is shown as a dashed line. BF biceps femoris, PA popliteal artery, PV popliteal vein, S sartorius, SM semi-membranous, T tibial nerve, VM vastus medialis

IPACK, introduced by the American Society of Regional Anesthesia in 2012, is a posterior analgesic method that involves the injection of an anesthetic solution into the space between the popliteal artery and the posterior capsule [26] (Fig. 10). After arising from the main trunks of the tibial and obturator nerves, the articular branches travel through a tissue space between the popliteal artery and the femur to innervate the posterior capsule of the knee. These articular branches can be blocked by infiltrating the tissue plane between the popliteal artery and the capsule of the knee (IPACK) with local anesthetic solution under ultrasound guidance. IPACK can selectively block only the innervation of the posterior knee joint while sparing the main trunks of the tibial and common peroneal nerves, thereby maintaining the sensorimotor function of the leg and foot. Thus, the use of IPACK for preserving motor analgesia for posterior knee pain is similar to ACB preserving motor analgesia for anterior knee pain.

There are few studies published in the literature evaluating the role of IPACK in pain management after total knee arthroplasty. Sankineani et al. [27] reported a significantly improved range of motion and walking distance as well as reduced visual analogue scale scores on the day of surgery for a group receiving ACB and IPACK compared with a group receiving ACB alone after total knee arthroplasty. Elliot et al. [28], in a study comparing patients who received ACB and IPACK after total knee arthroplasty with patients who received IPACK and FNB after total knee arthroplasty, reported that the former group had a reduced length of hospital stay, but showed no difference in visual analogue scale scores and opioid use. Kim et al. [29] compared a group who received PMDI during surgery with a group who received ACB and IPACK in addition to PMDI, and results showed that, in the latter group, the use of NRS and use of postoperative analgesics were significantly reduced compared with the group who only received PMDI. In the current study, and similar to the previous studies, the group that underwent ACB and IPACK showed more effective control of acute phase pain than the group undergoing ACB and PMDI after total knee arthroplasty.

Even though IPACK is known to be an effective posterior knee pain control method in some studies, there is still little research on the effective drug dosage, injection site and timing of IPACK. Thus, there is no consensus about the most effective administration methods (dosage, timing) using IPACK. One cadaver study of IPACK injection demonstrated that colored latex spread to the common peroneal nerve or tibial nerve in about 30–40% of cadavers after 10 ml of colored latex solution was injected to the popliteal fossa. Thus, they recommended that injection surrounding the middle genicular artery can consistently lead to effective IPACK block due to the predictable relationship between articular sensory nerves and this artery [30]. We did not observe any patients with muscle weakness using a 15-ml injection (more than the 10 ml mentioned above) via IPACK injection around the middle genicular artery. Further clinical study is needed for IPACK. To our knowledge, no comparative study of PMDI and IPACK with ACB has been performed in Korea or elsewhere, which is the aim of this study. We also used IPACK as an effective method for reducing posterior knee pain, similar to other studies.

This study has some limitations. First, this was a retrospective study with a small sample size. To minimize the influence of confounding factors, the surgeries were performed by a senior surgeon, and nerve blocks were performed by the same anesthesiologist using standard protocols. Differences in patient characteristics between the two groups may have affected the measured outcomes. Thus, we selected patients with similar baseline characteristics. Moreover, because this study was retrospective, power analysis was not performed. To obtain precise results, prospective studies performed with power analysis on a larger sample size are necessary. Second, this retrospective study included fewer males than females. The gender difference in tolerance to pain can affect outcomes. Thus, in our next study the gender ratio used will be the same. Third, to evaluate whether postoperative pain control method is effective, the range of motion and length of hospital stay should be included as well as pain assessment; however, our study did not evaluate anything other than that related to postoperative pain. Finally, general knee pain was used for the assessment of pain rather than posterior knee pain. Most of the patients were of an older age and had difficulty in expressing the exact pain site. Therefore, and inevitably, pain score was assessed with general knee pain. Unfortunately, this limitation may affect the results.

## Conclusions

Both IPACK and PMDI are effective in reducing initial postoperative pain after total knee arthroplasty. However, IPACK may be a better option than PMDI for controlling acute phase pain in patients undergoing total knee arthroplasty.

## Data Availability

Available.

## Abbreviations

ACB: Adductor canal block
BMI: Body mass index
FNB: Femoral nerve block
IPACK: Infiltration between the popliteal artery and the capsule of the knee
NRS: Numerical rating scale
PCL: Posterior cruciate ligament
PMDI: Periarticular multimodal drug injection
